# P-1832. Infection Prevention by Adequate Treatment of Iron Deficiency Anemia

**DOI:** 10.1093/ofid/ofae631.1995

**Published:** 2025-01-29

**Authors:** Eric Brownhill, Michael Rosenberg, Kenneth Rivlin

**Affiliations:** Jacobi Medical Center, New York City Health and Hospitals, Bronx, New York; Jacobi Medical Center, Bronx, New York; Jacobi Medical Center, New York City Health and Hospitals, Bronx, New York

## Abstract

**Background:**

Oral iron supplementation for iron-deficiency anemia (IDA) is well established as benign, but many practitioners limit its use in patients hospitalized for infectious causes. Nearly all human pathogens require iron, and must obtain it from the host, so administering iron during an infection is feared to exacerbate the infection. However, IDA itself is also associated with worse outcomes in infections, so iron stores must be balanced for optimal outcomes.

**Figure 1:**
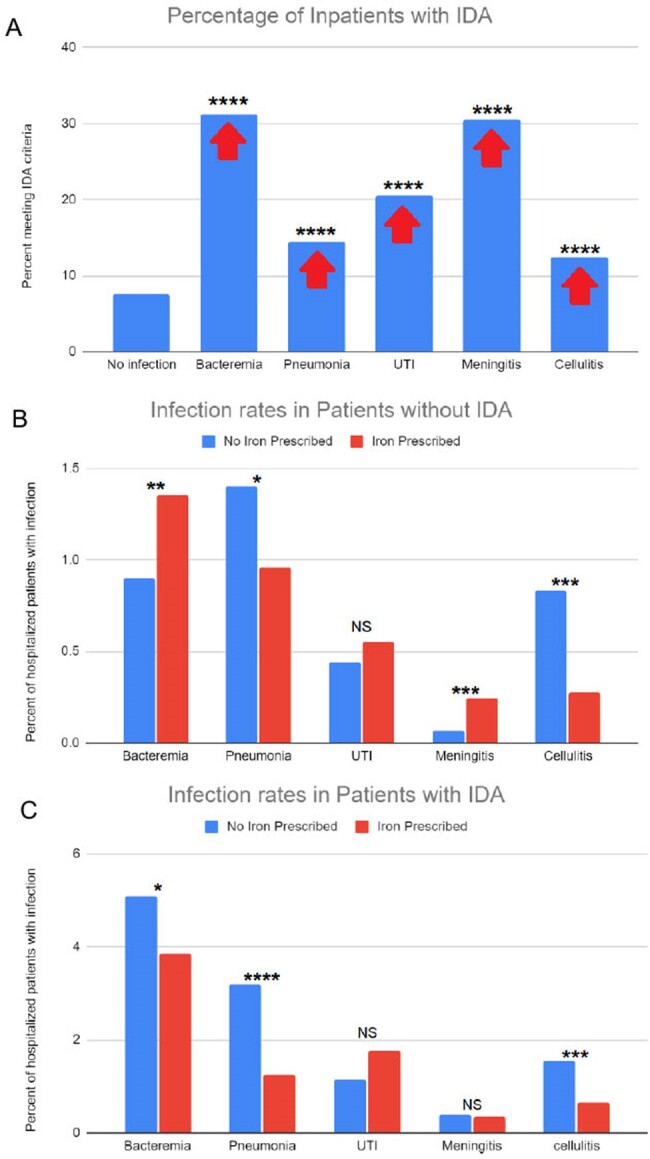
Comparison of IDA and iron usage in hospital admissions with infectious diagnoses. A. 7.6% of patients without an infectious diagnosis had IDA (abnormal MCV, Hgb, and RDW), compared to 31% admitted with bacteremia, 14% with pneumonia, 21% with UTI, 30% with Meningitis and 12% with cellulitis. B. Percentage of hospitalized patients with diagnosis of infection. In patients without IDA, prescribing iron is correlated with a significant increase in proportion of hospitalized patients with bacteremia and meningitis, a significant decrease in pneumonia and cellulitis, and no statistically significant effect on UTI. C. In patients with IDA, supplementation with iron is correlated with a significant reduction in proportion of hospitalized patients with bacteremia, pneumonia and cellulitis, but shows no statistically significant effect on UTI or Meningitis. *p < 0.05, **p < 0.01, ***p < 0.001, ****p < 0.0001 by chi-squared analysis, comparison to patients with no infection.

**Methods:**

Inpatient admission data for pediatric patients from multiple institutions, from 2020 to 2023 were retrospectively analyzed for infectious diagnoses on or during admission, IDA (defined by abnormal HgB, abnormal MCV, and abnormal RDW) and prescribed iron supplements. Of 91,029 hospital admissions, 7344 had IDA, of whom 1994 were prescribed iron. 3249 patients were prescribed iron with no IDA. Chi-squared analysis was performed on infection category: bacteremia, urinary tract infection (UTI), pneumonia, meningitis, and cellulitis, to find correlations between IDA, with or without iron supplementation, and frequency of hospitalization for each infection.

**Results:**

IDA correlates with an increased likelihood of all infections analyzed, on or during hospitalization. Iron supplementation in patients with no IDA correlated with increased bacteremia and meningitis, but not UTI, and correlated inversely with pneumonia and cellulitis. Treating IDA with iron correlated with decreased rates of pneumonia, cellulitis and bacteremia, but had no effect on meningitis or UTI. Correlations were consistent across all pediatric age ranges for both females and males.

**Conclusion:**

Our data demonstrate that IDA increases risk of severe infection, and iron overload (supplementation in absence of IDA) increases the risk of certain infections. However, iron supplementation actually decreases pneumonia and cellulitis rates at or during admission, regardless of IDA status. The skin and lungs are unique immune organs, in which macrophages (which require iron for antimicrobial functions) play key roles in homeostasis and infection control in epithelial barriers. Thus, based on these data, and supported by an understanding of macrophage innate immunity, iron repletion in IDA patients is critical to support immune function.

**Disclosures:**

**All Authors**: No reported disclosures

